# Seasonal Prevalence of Respiratory Pathogens Among Children in the United Arab Emirates: A Multicenter Cross-Sectional Study in the Pre-COVID-19 Era

**DOI:** 10.7759/cureus.45204

**Published:** 2023-09-14

**Authors:** Sara Salim, Handan Celiloglu, Farah Tayyab, Zainab A Malik

**Affiliations:** 1 College of Medicine, Mohammed Bin Rashid University of Medicine and Health Sciences, Dubai Healthcare City, Dubai, ARE; 2 Department of Microbiology, Mediclinic City Hospital, Dubai Healthcare City, Dubai, ARE

**Keywords:** mpcr, vaccination, enterovirus, rhinovirus, rsv, infection, pediatrics, respiratory, viruses

## Abstract

Background

Viral respiratory infections in children pose a significant burden on healthcare facilities globally. In the United Arab Emirates (UAE) these account for 15% of all healthcare encounters among children. However, the seasonal prevalence and molecular epidemiology of respiratory viral infections in the UAE remains unknown. We sought to determine trends in seasonal viral prevalence in order to monitor disease activity and optimize the timing of Respiratory Syncytial Virus (RSV) prophylaxis among high-risk infants in the UAE.

Methods

This cross-sectional multicenter study included children 0-18 years of age who presented to a large private healthcare group in Dubai, UAE, and had upper respiratory samples collected for multiplex polymerase chain reaction (mPCR) testing between January 1^st^ and December 31^st^, 2019. Sociodemographic, clinical, and molecular data were examined for children who tested positive for any pathogen on the mPCR panel.

Results

A total of three thousand and ninety-eight infants and children had mPCR assays performed during the study period, of which 2427 (78.3%) were positive for any respiratory pathogen. The median age of our sample population was 39 months and 56.8% were male. Emergency room was the most common site (34.7%) of sample collection and the vast majority of children presented with fever (85.3%). Rhinovirus/enterovirus was the most prevalent viral infection (45%) throughout the year and peaked in September, followed by Influenza (20.2%), and RSV (17.1%). RSV season, defined as an infection prevalence of >10%, occurred from August to December with a peak in October. Adenovirus (15.6%) infections peaked in June and accounted for 43% of hospitalizations in our study (*p*<0.05). Viral co-infections with RSV and rhinovirus/enterovirus were most common and observed in 19.9 % of children.

Conclusion

Rhinovirus/enterovirus is the most prevalent viral pathogen throughout the calendar year among the pediatric population in the UAE. RSV season begins earlier than reported in other countries regionally, hence RSV prophylaxis should be initiated in August to optimize protection among high-risk infants.

## Introduction

Viral respiratory infections are a leading cause of death in children under 5 years of age worldwide [1] and their etiology varies by geographic region [2,3]. Although many viral infections are limited to the upper respiratory tract, viral infections of the lower respiratory tract comprise more than 60% of acute lower respiratory illnesses in children, resulting in an enormous disease burden [4,5]. In countries with high coverage for routine infant and childhood vaccination, viral infections are responsible for the majority of febrile episodes in infants <3 months of age [6]. Studies using viral culture methods report that infants and preschool children in developed countries experience an average of 6-10 viral infections annually, while school-age children and adolescents experience 3-5 such illnesses every year [7]. Antibiotics are often prescribed for febrile illnesses in children, the vast majority of which are viral in origin, leading to antibiotic overuse. Respiratory syncytial virus (RSV) and influenza continue to cause considerable mortality and morbidity globally, despite the availability of RSV prophylaxis for high-risk infants, seasonal influenza vaccines, and antiviral agents [8].

Viral respiratory infections account for 15% of all healthcare encounters for children in the United Arab Emirates (UAE) [9]. Our study was conducted in the emirate of Dubai in the UAE, with a population of 3.35 million people from over 200 countries [10]. Dubai has a young population demographic, with 18% of its population aged 19 years or younger. Over the past decade, Dubai has rapidly emerged as a major global travel hub, welcoming nearly 16 million visitors annually [11]. Such a large-scale influx of global travelers is expected to impact the seasonality and prevalence of infectious diseases within a country. Hence understanding the trends in seasonal prevalence of respiratory viruses in the UAE will aid the clinician’s decision-making process and help limit antibiotic overuse in febrile children with viral symptoms. This is the largest study of its kind among children in the UAE and the wider Gulf region and aims to describe the disease burden, severity, and seasonality of respiratory viruses during a full calendar year in the pre-COVID-19 era.

This article was previously accepted for a poster presentation at the 20th Annual Pediatric Infectious Disease Society Virtual Research Conference, United States of America, on March 4, 2021 (Abstract #6).

*This article was previously posted to the In Review (Research Square) preprint server on April 7, 2023 *[12].

## Materials and methods

This cross-sectional study was conducted within a multidisciplinary university-affiliated healthcare network, which comprises three large tertiary-care hospitals and ten outpatient clinics in Dubai, UAE. These facilities are served by a centralized microbiology laboratory which processes multiplex polymerase chain reaction (mPCR) samples from all sites within the network and is also a referral laboratory for non-network healthcare facilities. Our study population included children who presented to any medical facility served by the centralized microbiology laboratory between January 1st and December 31st, 2019, and tested positive for any pathogen on nasopharyngeal samples by mPCR testing. All children, birth to 18 years, who after clinical evaluation by the consulting pediatrician were deemed to have symptoms of a viral illness necessitating mPCR testing constituted the study population. We excluded children if they were older than 18 years, or if samples were collected for testing by any methods other than mPCR. 

Nasopharyngeal samples were collected by physicians or nurses using medium-sized flocked fiber tip swabs and immediately placed in universal viral transport media for transport and processing at the centralized microbiology laboratory. Samples were tested using the FilmArray™ multiplex PCR (Biomerieux, Askim, Sweden) which tests for 17 viruses and three bacteria commonly responsible for upper respiratory tract infections, and has a high manufacturer-reported sensitivity (95%) and specificity (99%) [13]. This is a closed, fully automated system that extracts nucleic acids and runs the PCR cycle. The FilmArray™ software analyzes and interprets the assay results and a final report is generated within one hour of sample processing. This test report is reviewed and authorized by the microbiology laboratory staff before releasing it to the patient’s electronic medical records. The microbiology laboratory is fully staffed and runs 24 hours a day, 7 days a week. It is accredited by the College of American Pathologists (CAP) and Joint Commission International (JCI) and holds the ISO-15189 certification.

Data was collected for patient-related clinical, demographic, and epidemiological variables. In addition, we collected information on the patient’s site of presentation for testing, i.e., inpatient, clinic, emergency room, or external sample, and receipt of the seasonal influenza vaccine [14]. Molecular data for pathogen identification, co-infection, and calendar month of infection were also collected. Data was analyzed using the Statistical Package for Social Sciences (SPSS) software (version 25.0; IBM Corp., Armonk, USA). Frequencies with proportions were reported for categorical variables and means with standard deviations (SD) were reported for continuous variables. Association between categorical variables was tested by the chi-square and Fischer Exact test when appropriate. *P* value ≤0.05 was considered statistically significant in all analyses. 

In all cases, parents or caregivers signed a consent form while registering their child for treatment at the medical facilities. Informed consent was waived since the nasopharyngeal samples were collected during the provision of routine clinical care. This study was reviewed and approved by the Institutional Review Boards (IRBs) of Mediclinic Middle East and Mohammed Bin Rashid University of Medicine and Health Sciences (MBRU) Student Research Projects (SRP) committee (Approval No. MBRU-IRB-SRP-2020-13). 

## Results

**Figure 1:**
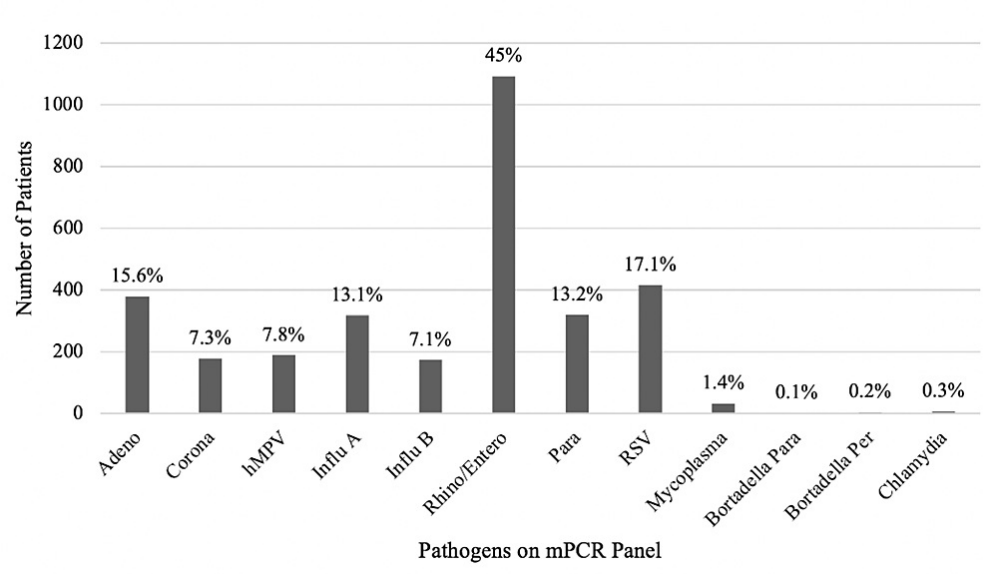
Prevalence of Respiratory Viral Pathogens: Results of 2427 Patients With Positive PCR Adeno – Adenovirus; Corona – Coronavirus; hMPV – Human Metapneumovirus; Influ A – Influenza A; Influ B – Influenza B; Rhino/Entero – Human rhinovirus/Enterovirus; Para – Parainfluenza virus; RSV – Respiratory Syncytial Virus; Mycoplasma – *Mycoplasma pneumoniae*; Bordetella para – *Bordetella parapertussis*; Bordetella Per – *Bordetella pertussis*; Chlamydia – *Chlamydia pneumoniae*; PCR – polymerase chain reaction

A total of three thousand and ninety-eight children had mPCR assays performed during the study period, of which 2427 (78.3%) were positive for any respiratory pathogen. All children with mPCR assay positive for at least one respiratory pathogen were analyzed for molecular epidemiology and seasonality. Of the 2,427 children positive for any respiratory pathogen, 688 (28.3%) were co-infected with two or more pathogens resulting in a total of 3115 positive samples. Overall, human rhinovirus/enterovirus (HRV) was the most prevalent pathogen, followed by Influenza, RSV, and adenovirus (Figure 1). 

**Figure 2:**
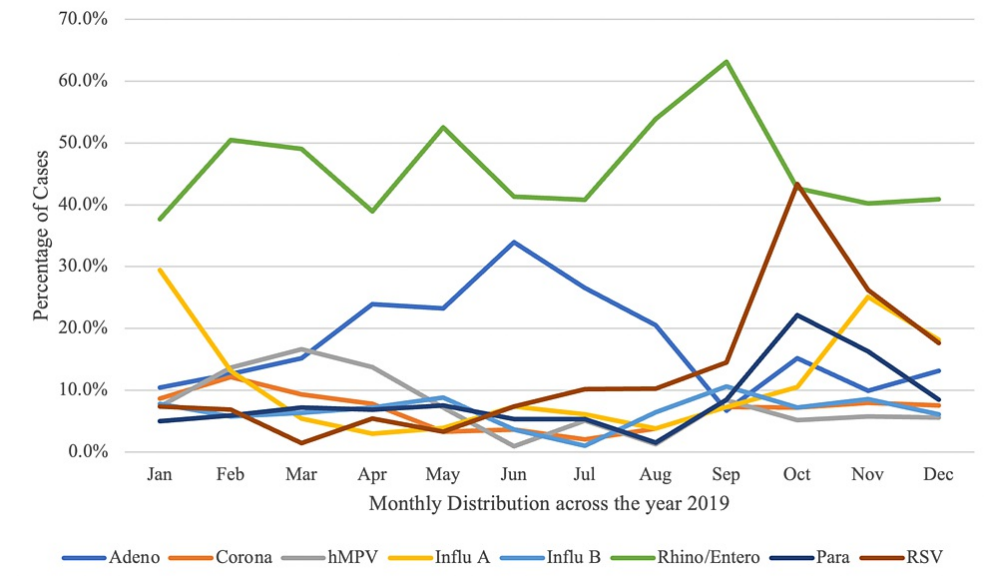
Monthly Distribution of Respiratory Viral Pathogens* *3115 positive samples from 2427 children. Adeno – Adenovirus; Corona – Coronavirus; hMPV – Human Metapneumovirus; Influ A – Influenza A; Influ B – Influenza B; Rhino/Entero – Human rhinovirus/Enterovirus; Para – Parainfluenza virus; RSV – Respiratory Syncytial Virus; Mycoplasma – *Mycoplasma pneumoniae*; Bordetella para – *Bordetella parapertussis*; Bordetella Per – *Bordetella pertussis*; Chlamydia – *Chlamydia pneumoniae*.

Among children with viral co-infections, 137 were simultaneously infected with HRV and RSV (19.9%), 112 with HRV and adenovirus (16.2%); and 50 children (7.2%) were co-infected with HRV and coronaviruses (229E, KHU1, NL63 and OC43). Adenovirus was the most commonly reported virus among hospitalized children (43%, p 0.005) but accounted for only 15.6% of all infections among children. Adenovirus demonstrated a high prevalence in the summer with a peak in June, contributing to pediatric hospitalizations during the summer months (Figure 2).

HRV was found to be highly prevalent throughout the year, with a peak in September. RSV season, defined as an infection prevalence of >10%, began in August and petered out by December with a distinct peak occurring in October. Influenza infections predominated during the winter months, as expected, with 491 children testing positive for influenza, comprising 20.2% of all samples positive for any respiratory virus on mPCR. Influenza A was the predominant strain among children testing positive for influenza (64.8%). During our study period, influenza A peaked in January during the 2018-2019 flu season, followed by a second peak in November during the 2019-2020 flu season. Influenza B, on the other hand, showed a much lower prevalence throughout the calendar year, demonstrating a rise in prevalence in early August and hitting a peak in September.

**Figure 3:**
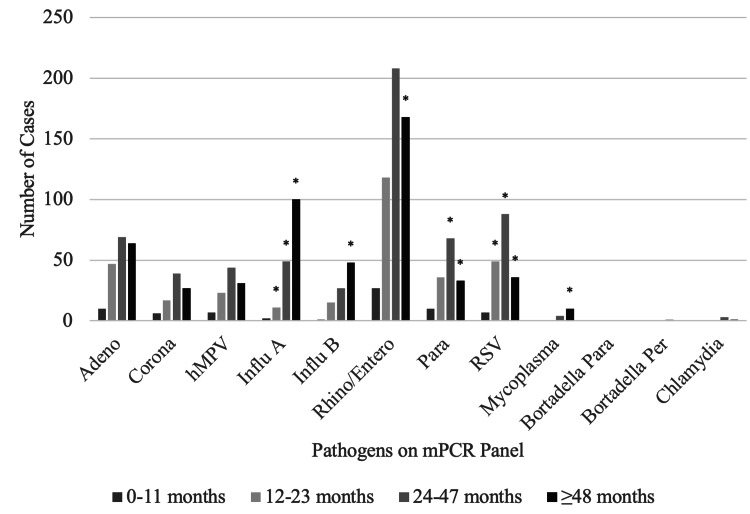
Age-Specific Distribution of Respiratory Viral Pathogens *p <0.05 Adeno – Adenovirus; Corona – Coronavirus; hMPV – Human Metapneumovirus; Influ A – Influenza A; Influ B – Influenza B; Rhino/Entero – Human rhinovirus/Enterovirus; Para – Parainfluenza virus; RSV – Respiratory Syncytial Virus; Mycoplasma – *Mycoplasma pneumoniae*; Bordetella para – *Bordetella parapertussis*; Bordetella Per – *Bordetella pertussis*; Chlamydia – *Chlamydia pneumoniae*.

Data for age was available for 1177 children, and age was categorized into clinically-relevant groups: 0-11 months (n= 52, 4.4%), 12-23 months (n= 241, 20.4%), 24-47 months (n= 444, 37.7%), and ≥ 48 months (n= 440, 37.3%). Human rhinovirus/enterovirus was the most common virus identified in patients in all age groups, accounting for 43.2% of positive cases (p 0.01) (Figure 3). 

The median age of our population was 39 months, and boys were marginally over-represented in our study (56.8%). The vast majority of our patients were expatriate children (83.8%), which is reflective of the population mix in Dubai. The most frequent site of testing was the emergency room (34.7%), followed by inpatient units (27.5%) and outpatient clinics (19.7%). Samples from non-network facilities comprised a small proportion of positive samples (16.3%) in our study.

**Figure 4:**
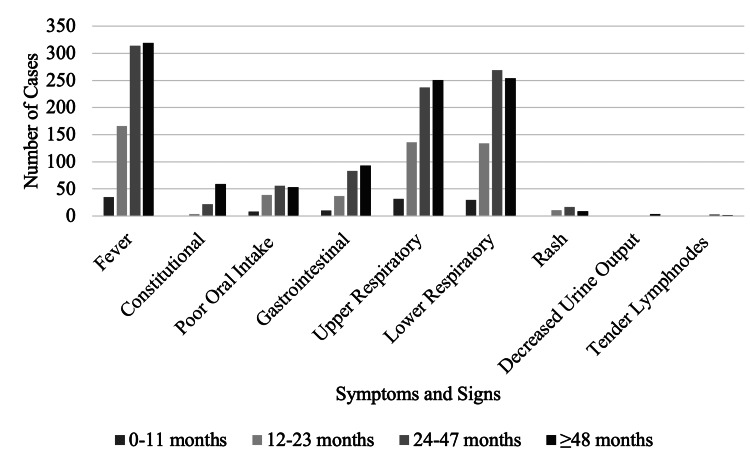
Age-Specific Distribution of Signs and Symptoms

Fever was the most common presenting symptom in all age groups (85.3%) followed by lower (70.3%) and upper respiratory symptoms (67%). Age-specific distribution of symptoms is given in Figure 4. 

Up to 35.9% of the children in our study had another infection associated with their presenting respiratory illness. Streptococcal pharyngitis was the most common bacterial co-infection, while urinary tract infections (UTIs) were infrequently reported. One hundred and twelve children infected with Rhinovirus/Enterovirus were co-infected with Streptococcal pharyngitis, making this the most frequent bacterial co-infection in our study.

Our patient population was healthy, with only 12.7% of children reporting a significant past medical history. Asthma (9.6%), eczema (1.6%), and febrile convulsions (1.5%) were the most commonly reported comorbid conditions. Rates of seasonal influenza vaccination among our patients were low (10.7%). UAE nationals represented 26.5% of children with asthma (p 0.036). However, asthma was not a major risk factor for pneumonia in our study, with only 12.7% of children with pneumonia reporting a past history of asthma. 

## Discussion

This is the largest study in the UAE and the wider Gulf region reporting the incidence and molecular epidemiology of respiratory pathogens causing acute respiratory infections among children. We report an overall positivity rate of 78.3% on mPCR testing, which is much higher than previously reported in the UAE by Jeon et al. among a mixed-age population [8]. Our findings are consistent with high rates of viral positivity reported among young children with respiratory symptoms in the region [15, 16]. This is reflective of the higher prevalence of viral respiratory infections in children compared to adults worldwide. 

HRV is a very common respiratory virus globally. In our study, HRV was prevalent throughout the year and was by far the most common virus reported among children, followed by Influenza then RSV. This is similar to other pediatric data from the UAE [8], but different from Saudi Arabia where RSV was the most common viral pathogen reported among children, followed by HRV [16,17]. The high frequency of HRV infections reported among studies of children in the UAE could be explained by a combination of factors, including healthcare-seeking behaviors of parents with young febrile children, the widespread availability of mPCR testing and accessibility of children with mild viral illnesses to outpatient clinics and emergency rooms, resulting in an overestimation of HRV in our pediatric population compared to other countries in the region. HRV is recognized as a major viral trigger for asthma exacerbations in children and adults owing to various viral, host, and immune factors [18]. This was first reported by Minor et al. almost fifty years ago when they described a clear association between HRV infections and asthma exacerbations [19]. Asthma was the most frequent chronic disease reported in 9.6% of our total sample population. UAE nationals represented 16% of our study population and constituted 26.5% of children with asthma in our cohort (p 0.036) - a disproportionately high prevalence. Our findings highlight the need for children with asthma living in the UAE to be closely followed by their pediatrician to ensure good asthma control in order to limit exacerbations, especially during trigger months of the year. 

Viral co-infection, or the simultaneous detection of two or more viral pathogens on mPCR in a single patient may result from true infection, asymptomatic colonization, or viral shedding [20]. However, making the distinction is difficult in the context of an acute illness. Viral co-infections were noted in up to a third of our patients. This is comparable to other PCR-based studies which have reported rates of 11% to 39% among children [20-24]. Among our patients, those with influenza were unlikely to be co-infected, while HRV and RSV tended to be found together frequently. In contrast, other investigators have reported RSV and adenovirus occurring as single infections [20]. Esposito et al., also reported a high rate of viral co-infections (29%), mostly dual infections with RSV and HRV, among children in Italy [25]. These results suggest that certain viruses likely facilitate infection or colonization with other viruses in the same patients. This is supported by a study from the United States which demonstrated that rhinovirus reduces replication of the other viruses during a co-infection, while parainfluenza virus is suppressed in the presence of other viruses [26].

Urinary tract infections (UTIs) are the most commonly reported secondary serious bacterial infection (SBI) in infants and children with viral bronchiolitis [27]. These have been described most frequently with RSV and influenza infections [28]. However, other studies have reported UTIs in infants with bronchiolitis secondary to other viral etiologies as well [29]. Interestingly, children with mPCR positive for respiratory viruses were rarely found to have a UTI in our study and need further research. Streptococcal pharyngitis was the most common associated infection in our study population of children with a viral illness. This is likely an overdiagnosis since a positive streptococcal antigen test or throat culture in the context of a viral illness is reflective of colonization rather than true infection. Unfortunately, this leads to antibiotic overuse and contributes to antimicrobial resistance in the community. This finding highlights the urgent need for physician education and awareness regarding performing streptococcal antigen testing and throat cultures only in patients who present with sore throat and fever without concurrent viral symptoms, in accordance with international guidelines [30]. 

Fever and respiratory symptoms were the most common clinical features across all age groups among our patients. Interestingly, upper and lower respiratory symptoms occurred in a similar frequency among our patients. These similarities in clinical presentations across various viral etiologies make it impossible to diagnose specific viral pathogens on clinical grounds. Data on the seasonality of viruses can help clinicians make informed decisions in settings where molecular detection methods are unavailable. Other studies have suggested that a possible interference between different viruses might also contribute towards viral seasonality [31,32]. For instance, rhinovirus infections trigger a strong interferon response, therefore creating a non-favorable environment for other viruses during peak rhinovirus season [33-35]. We found seasonality in prevalence for most viruses in the UAE. This is in parallel with studies from Germany [31] and Sweden [36], which have demonstrated peaks of viral prevalence during the start of autumn, winter, and rainy seasons. Rhinovirus is also the most common viral pathogen throughout the year in Sweden [36], which is comparable to our findings. Observational studies have reported that the seasonality of many respiratory viruses is influenced by the weather. An example is RSV, which thrives in cooler temperatures, hence RSV infections increase with the start of autumn [37-39]. Interestingly, an inverse relationship has been found between rhinovirus infections and increased humidity [37,38]. However, HRV infections peaked in September in our study which correlates with high levels of humidity of up to 60% in the UAE. Whether this September peak is triggered by the start of the academic school year for children in the UAE needs further exploration. 

The overall prevalence of respiratory viruses declined with age in our study, which is similar to reports from other regions. We found no significant correlation between influenza vaccination and reduced hospitalization rates, likely due to the small number of children in our study who had received the seasonal influenza vaccine. Hence it is difficult to derive any meaningful results on the impact of influenza vaccination on health outcomes and needs further exploration. Our findings also highlight the need to improve public awareness in order to increase rates of influenza vaccination, especially among children who are at increased risk of influenza-related complications [40]. 

To our knowledge, this is the largest study exploring the seasonality of respiratory infections among children in the UAE. Our molecular epidemiology data for all children presenting during the full calendar year has important public health implications. Surveillance and monitoring of the annual seasonal characteristics of respiratory viruses are critical in predicting and detecting epidemics and pandemics. Our findings will help in early viral diagnosis, prevention of unnecessary antibiotic usage through patient and physician education, early initiation of antiviral treatment in high-risk patients, and initiating RSV prophylaxis and seasonal influenza vaccination in high-risk infants and children in a timely manner. 

An important limitation of our study is that our samples were collected from children in Dubai, UAE, thus limiting the generalizability of our study findings to the wider Gulf region. Another potential limitation is that our findings are reflective of viral prevalence and seasonality in the pre-COVID-19 era. It can be assumed that the restrictions in travel, social interaction, and widespread masking brought on by the pandemic may have resulted in significant shifts in viral distribution regionally and globally. This highlights the need for ongoing surveillance and tracking of viral respiratory infections among children and adults in a post-pandemic world. It will be interesting to repeat our study in the post-COVID-19 era to explore shifts in the viral epidemiology and prevalence within the same population. 

## Conclusions

In conclusion, this large multicenter study provides a valuable snapshot of viral epidemiology among children throughout a full calendar year in the UAE. Physician awareness about the high prevalence of viral pathogens among children presenting with febrile illnesses and peaks of viral activity during certain months will help limit unnecessary streptococcal testing and antibiotic prescribing, leading to a reduction in antibiotic overuse and misuse. Furthermore, our study will help physicians and public health bodies predict the beginning of RSV and influenza seasons, helping to initiate timely prophylaxis and vaccination in high-risk infants and children. 
